# Quality of Root Canal Obturation Performed by Senior Undergraduate Dental Students

**Published:** 2013-12-24

**Authors:** Saeed Moradi, Maryam Gharechahi

**Affiliations:** a *Dental Material Research Center, Department of Endodontics, Dental School, Mashhad University of Medical Sciences, Mashhad, Iran; *; b * Dental Research Center, Department of Endodontics, Dental School, Mashhad University of Medical Sciences, Mashhad, Iran*

**Keywords:** Dental Radiography, Endodontics, Root Canal Obturation, Root Canal Therapy, Undergraduate Medical Education

## Introduction

**Introduction**: There is a direct relationship between the quality of root canal obturation and success of endodontic therapy. The aim of the present study was to assess the quality of canal obturation performed by undergraduate dental students at the Faculty of Dentistry, Mashhad University of Medical Sciences, Mashhad, Iran.** Materials and Methods:** Records of 200 endodontically treated teeth from patients who were visited by undergraduate students between 2009 and 2010, at the Endodontic department, Faculty of Dentistry, Mashhad University of Medical Sciences, were randomly selected for evaluation. Periapical radiographs of all treated teeth were assessed in terms of canal obturation quality (*i.e.* adequate density and length). Statistical analysis of data was carried out using SPSS software and chi-square test. The Statistical significance level was set at *P*=0.05.** Results:** Forty-five percent of teeth fulfilled the criteria of an acceptable root canal obturation. Adequate length and density of root filling was found in 89% and 34% of teeth, respectively. There was a significant difference between maxillary and mandibular teeth regarding the length of root canal obturation (*P*=0.004). A significant difference was observed between molars and other tooth types. The frequency of root canals with an acceptable filling was significantly greater in the anterior teeth compared to premolars or molars. **Conclusion:** The technical quality of root canal treatment performed by undergraduate dental students was found to be less than ideal.

## Introduction

Preservation of the natural dentition has become more popular in contemporary society [1]. Therefore, endodontic therapy is becoming an increasingly routine part of general dental practice [2]. Success of root canal treatment has been reported to be 53-94% [3, 4].

The methods for assessment of endodontic treatment outcome are mostly based on radiographic evaluation only [5-7], combination of radiographic and clinical evaluation [8-10] or histological examination, in case of treatment failures [11, 12]. The quality of root canal treatment performed by general practitioners in different populations has also been extensively investigated in many studies, the results of which have shown high percentage of inadequate root canal treatment[7, 13-15]. The reasons are complex and may be related to the different endodontic teaching methods at dental schools [16].

Some of the problems in endodontic teaching may be attributed to limited time allocated to endodontics, poor staff-to-student ratios and the fact that many endodontists have not taken the teaching skills [17].

Students are taught endodontics within five levels, at the Faculty of Dentistry, Mashhad University of Medical Sciences, Iran. A preclinical course is presented in the third year within two semesters, during which students are trained to perform root canal treatment on human extracted teeth. They are expected to complete the root canal treatment (RCT) of at least 5 anterior teeth, 2 bicuspids and 4 molars. The other three courses are clinical practice, where senior students of 4th, 5th and 6th years, are expected to perform endodontic treatment on a variety of teeth belonging to the patients diagnosed to require RCT. During the 4th year, the students perform the RCT of 6 anterior teeth and 2 bicanaled premolars. In 5th year, they have to complete the RCT of 8 molars, one premolar, and carry out one retreatment within the two terms. And finally, in 6th year, during one endodontic course root canal treatment of 3 molars should be carried out. So, the fifth year seems to be the appropriate time for evaluating the technical quality of root canal therapy by undergraduate students.

**Table 1:** The criteria for the assessment of radiographic quality of root canal filling

**Parameter**	**Criteria**	**Definition**
**Length of root canal filling**	Adequate	Root filling ending ≤2 mm from radiographic apex
	Over-filling	Root filling beyond the radiographic apex
	Short-filling	Root filling >2 mm from radiographic apex
**Density of root canal filling** **quality **	Adequate	No voids present in the root filling or between root filling and root canal walls
	Inadequate	Voids present in the root filling or between root filling and root canal walls
	Acceptable	Adequate length and density

**Table 2:** Distribution of teeth in maxillary and mandibular arches

**Tooth type**	**N**	**%**
**Maxillary**	112	56
**Maxillary anterior teeth**	60	30
**Maxillary premolars**	27	13.5
**Maxillary molars**	25	12.5
**Mandibular**	88	44
**Mandibular anterior teeth**	1	0.5
**Mandibular premolars**	20	10
**Mandibular molars**	67	33.5
**Total**	200	100

Eleftheriodis *et al.* evaluated root canal obturation by 4th and 5th year undergraduate students [18]. Balto *et al.* [19] and K-fir *et al.* [20] in separate studies assessed the quality of root canal therapy carried out by fifth-year undergraduate students. Such studies are necessary in order to assess the effectiveness of medical education and dental care and also help with planning of future dental training strategies.

The aim of this observational study was to evaluate the technical quality of root canal obturation using radiographs of teeth treated by fifth-year undergraduate dental students at Endodontic department, Faculty of Dentistry, Mashhad University of Medical Sciences, Mashhad, Iran.

## Material and Methods

Records of 245 patients who had received endodontic treatment by fifth-year undergraduate students at Faculty of Dentistry, Mashhad University of Medical Sciences, from 2009 to 2010, were selected and evaluated. Records that did not include pre- and post-operative periapical radiographs or those with poor radiographic quality besides the records of patients with incomplete endodontic treatment, were excluded. The final sample consisted of 200 documents of endodontically treated teeth. Among these, 61 were anterior teeth while 47 and 92 teeth were premolars and molars, respectively. All the RCTs had been performed by fifth-year undergraduate students. An aseptic technique with rubber dam isolation had been applied in all the cases and working lengths was determined by means of radiographs. All the teeth had been instrumented with passive step-back technique using stainless steel K-files (Dentsply, Tulsa, Ok, USA) with 0.02 taper and irrigated with 2.5% sodium hypochlorite solution. Root fillings had been carried out with lateral compaction technique using gutta-percha (Ariadent, Tehran, Iran) and AH-26 sealer (Dentsply Maillefer, Tulsa, OK, USA). All the teeth had been temporarily filled and refereed for permanent restoration.

All the treatment steps were conducted under the supervision of teaching staff and postgraduate students of the department with an average staff-to-student ratio of 1:5. Evaluation of the technical quality of RCT was based on the immediate postoperative radiograph of each case.

For the present assessment, the quality of root canal obturation was evaluated according to the criteria of Barrieshi–Nusair *et al.* , and the final radiographs were examined by two independent investigators using a with a few selected cases which were not included in the study[16].

An evaluation form was designed to record the information gathered from the immediate postoperative radiographs. The method of viewing the radiographs was standardized; they were interpreted in a dark room using an illuminated viewer box (Dentsply Rinn Corp. Elgin, IL, USA) with magnification (2×) whilst mounted in a cardboard slit to block off ambient light emanating from the viewer. Measurements were recorded using a transparent ruler of 0.5 mm accuracy. The results were compared and a final consensus was agreed upon. In case of disagreement, a third investigator was asked to evaluate the radiograph and a final agreement was reached. Each root was scored individually and the tooth was considered as a unit. In multi-rooted teeth, the highest score of all the roots was attributed to the tooth and ultimately, failure of one root was considered the failure of the tooth as a whole. The quality of endodontic treatment was determined by the length of root canal obturation in relation to the radiographic apex and also the density of the obturation based on the presence of voids (Table 1). Acceptable obturation quality was defined as adequate length and density with the absence of any procedural error.

Statistical analysis of data was carried out using SPSS software, version 7.5 (SPSS Inc., Chicago, IL, USA) and the chi-square test. Statistical significance was set at *P*<0.05.

**Table 3:** Overall quality, length, and density of root canal filling

	**Quality**		**Length**			**Density**	
**n**	**Acceptable**	**Unacceptable**	**Adequate**	**Short-filling**	**Over-filling **	**Acceptable**	**Unacceptable**
**200**	90 (45%)	110 (55%)	178(89%)	15(7.5%)	7(3.5%)	69(34.5%)	131(65.5%)

**Table 4:** Quality, length, and density of root canal filling in relation to teeth position

		**Quality**		**Length**			**Density**	
**Arch**	**n**	**Acceptable**	**Unacceptable**	**Adequate**	**Short-filling**	**Over-filling**	**Acceptable**	**Unacceptable**
**Maxillary**	112	51(45.5%)	61(54.5)	106(94.6)	3(2.7%)	3(2.7%)	39(34.8%)	73(65.2%)
**Mandibular**	88	39 (44.3%)	49 (55.7%)	72(81.8%)	12(13.6%)	4(4.5%)	30(34.1%)	58(65.9%)

**Table 5:** Quality, length, and density of root canal filling according to teeth type

		**Quality**		**Length**			**Density**	
**Tooth type**	**n**	**Acceptable**	**Unacceptable**	**Adequate**	**Short-filling**	**Over-filling**	**Acceptable**	**Unacceptable**
**Anterior**	61	40(65.6%)	21(34.4%)	60(98.4%)	0(0%)	1(1.6%)	21(34.4%)	40(65.6%)
**Premolar**	47	29(61.7%)	18 (38.3%)	44(93.6%)	2(4.3%)	1(2.1%)	15(31.9%)	32(68.1%)
**Molar**	92	21 (22.8%)	71 (77.2%)	74(80.4%)	13(14.1%)	5(5.4%)	33(35.9%)	59(64.1%)

## Results

The teeth were classified according to their location in the arches. The frequencies of teeth examined in this study are shown in Table 2. There were 112 maxillary and 88 mandibular teeth. Quality of root canal filling (*i.e.* length, and density) is shown in Table 3. Ninety teeth (45%) fulfilled the criteria of an acceptable root canal filling. Adequate length of the root filling was found in 89% of teeth, while 7.5% were underfilled and 3.5% were overfilled. Adequate density was found in 34% of teeth.

There was no statistically significant difference between maxillary and mandibular teeth in terms of the quality of root fillings (*P*=0.864). However, there was a significant difference between maxillary and mandibular teeth in relation to the length of root canal obturations (*P*=0.004). Adequate length of root filling was found in 94.6% of maxillary and 81.8% of mandibular teeth. A total of 13.6% of mandibular and 2.7% of maxillary teeth were underfilled, while 2.7% and 4.5% of the teeth were overfilled in maxilla and mandible, respectively. Adequate density was found in 34.8% of maxillary and 34.1% of mandibular teeth (Table 4).

There was a relationship between tooth type and the quality of root filling. A significant difference was observed between molars and other tooth types (*P*=0.000). The frequency of root canals with an acceptable obturation was significantly higher in anterior teeth (40%) compared to premolars (29%) or molars (21%) (Table 5).

## Discussion

This study aimed to evaluate the quality of root canal treatment carried out by senior undergraduate students at Faculty of Dentistry, Mashhad University of Medical Sciences. Postoperative periapical radiographs were used for assessment. The quality of root canal obturation was evaluated according to the criteria of Barrieshi–Nusair *et al.* [16].

Studies evaluating the radiographic quality of root canal treatment have mostly been based on the evaluation of the length and the density of root canal obturation [21-24]. The results of the present study demonstrated adequate quality of root fillings in 45% of teeth, similar to the results of a study performed by Barrieshi-Nusair* et al*. [16]. Such frequency was lower than the frequency reported by Benenati *et al.* (91.05%) [25], Al-Yahya *et al.* (76%) [26], Lynch (63%) [27], and Eleftheriadis and Lambrianidis (55%) [18]. Also, the results were higher than those reported by Hayes *et al.* which was 13% [28].

The results of this study showed that quality of root canal obturation was less than ideal. The reasons for this fact are complex and may be related to the endodontic teaching strategy undertaken at dental schools [16].

The quality of maxillary and mandibular root fillings was the same in this study. The frequency of teeth with an acceptable root canal obturation was significantly higher in anterior teeth (40%) compared to premolars (29%) and molars (21%) (*P*=0.000). Such results are consistent with the findings of Boucher *et al.* [29] and Eleftheriadis and Lambrianidis [18], who reported that the technical quality was acceptable more often in anterior teeth. This may be explained partly by the anatomy of these teeth.

The percentage of root canal fillings with adequate length was 89% in the present study. Although it is difficult to compare these results with those of other studies, the percentage of root fillings with adequate length was higher when compared with those reported by Er *et al.* (69.6%) [30], Chueh *et al.* (61.7%) [31] and Barrieshi-Nusair *et al.* (76.7%) [16]. However, estimation of the root filling length was probably not reproduced correctly in all the radiographs because the undergraduate students use the bisecting-angle technique to take the postoperative radiographs. Forsberg demonstrated that root canal fillings are projected shorter (*i.e.* more coronally), on the radiographs exposed with the bisecting-angle technique than with the paralleling technique [32].

In the present study, underfillings were found in 7.5% of the teeth. The highest percentage of underfilling was found in mandibular molars. This finding concurs with those of studies by Barrieshi-Nusair *et al.* [16] and Er *et al.* [30] and may be explained by the anatomy of these teeth, *i.e.* multi-canalled roots and their curvature and also difficult access to posterior teeth that make their root canal treatment more challenging for students.

In this study overfilling was found in 3.5% of the teeth. The higher percentage of overfillings reported in other studies may be attributed to the higher incidence of teeth with preexisting periapical radiolucency in these studies [10, 19, 30]. These lesions can result in resorption and destruction of the apical constriction and this loss may have influenced working length control by undergraduate students.

Inadequate density of root canal obturation may lead to failure of root canal treatment because of microleakage along the root filling [23]. Eriksen and Bjertness reported the incidence of apical periodontitis to be higher in root-filled teeth with inadequate densities [33]. The results of the present study indicated that adequate density occurred in 34.5% of cases. Such frequency was consistent with the study of Balto *et al.*, who reported adequate density in 35% of teeth [19]. On the contrary, it was lower than the values reported by Yoldas *et al.* (64%) [34] and Er *et al.* (53%) [30]. However, it is difficult to compare these studies as a result of differences in their sample size.

In Mashhad Faculty of Dentistry, passive step-back instrumentation using conventional stainless steel files and cold lateral condensation are taught to undergraduate dental student. These techniques are the most widely taught and used techniques in dental schools [35]. A number of schools have incorporated the use of rotary nickel-titanium (NiTi) instruments in their undergraduate curricula [36]. Some studies have shown that when dental students use either hand or rotary NiTi instruments, canals are prepared with less procedural errors and more successful treatment results are achieved compared to the use of conventional stainless steel instruments [37-40].

In our school, preclinical endodontics is taught in two academic terms. Each term lasts 4 months with an allocation of 3 h per week at the phantom-head laboratory. This short time tends to limit students’ preclinical training in endodontics with consequent problems during clinical practice. Some investigators, who have evaluated the undergraduate endodontic teaching, have reported similar teaching problems [17, 36, 41].

To sum up, in order to improve the technical quality of root canal treatment performed by undergraduate dental students, the endodontics curricula have to be revised. Thus, the time of training of the students at the pre-clinic and clinic has to be extended, and subsequently the clinical requirements for endodontics have to be increased, so that the student will be given more time to treat more cases. The clinical training courses have to be arranged to provide the students with proper skills in endodontics, starting with the basic principles in clinical practice.

## Conclusion

According to the results of this observational study, the technical quality of root canal treatment performed by undergraduate dental students was found to be less than 50%. Thus, revision of training courses for the students at the pre-clinic and clinic is suggested.
